# Epidemiology and associated factors of atopic dermatitis in Malagasy children

**DOI:** 10.1186/s13223-019-0398-2

**Published:** 2020-01-06

**Authors:** F. A. Sendrasoa, I. M. Ranaivo, N. H. Razanakoto, M. Andrianarison, O. Raharolahy, V. T. Ratovonjanahary, M. Sata, M. F. Rakotoarisaona, L. S. Ramarozatovo, F. Rapelanoro Rabenja

**Affiliations:** 1 Department of Dermatology, University Hospital Joseph Raseta Befelatanana, Antananarivo, Madagascar; 2 Department of Dermatology, University Hospital Morafeno, Toamasina, Madagascar

**Keywords:** Associated factors, Atopic dermatitis, Antananarivo Madagascar, Prevalence

## Abstract

**Background:**

Little is known about the epidemiology and associated factors of childhood AD in the markedly different, low-income, tropical environment like Madagascar.

**Methods:**

We aim to assess the epidemiology and associated factors of AD in individuals fewer than 15 years of age in Antananarivo Madagascar. It was a retrospective and descriptive study over a period of 7 years (2010 to 2016) in children 6 months to 14 years in the Department of Dermatology, Joseph Raseta Befelatanana Antananarivo Madagascar. The diagnosis of AD was based on clinical data.

**Results:**

The prevalence of AD was 5.6% in children aged 6 months to 14 years. The details of 151 cases of atopic dermatitis were analyzed. The mean age of patients was 4 years. There was a female preponderance (sex ratio: 0.7). A family history of AD was noted in 56 cases (37%). No association between breast-feeding and AD was found. The age of onset of AD was before the age of 3 months in 7.5% and between 6 months to 5 years in 70%. Children born in March (dry season) had the highest risk of AD. Consultations for AD increased during the winter (from July to October; p = 0.005). However, the prevalence of AD was similar in urban and rural areas.

**Conclusion:**

Weather may have an impact on the prevalence of atopic dermatitis in Madagascar. No significant correlation was found between the duration of breastfeeding and AD, as well as urbanization.

## Introduction

Atopic dermatitis (AD) is a chronic inflammatory skin disorder that is characterized by intense itching and recurrent eczematous lesions. AD affects up to 20% of children and 3% of adults; latest global data shows increases in its prevalence [1]. In Africa, the prevalence of AD ranges from 4.7% to 23%. The onset of AD is usually between 2 and 6 months of age, although it can begin at any age [2]. It was previously thought that it resolved by adulthood in most cases, but evidence suggests that it is a chronic condition that may persist into adulthood [3].

Several factors such as environmental exposures (use of personal care products, climate exposure, pollution, food…) and genetics attribute to the increased global prevalence of AD in predisposed individuals [4]. We aim to describe the prevalence and factors associated of AD in Antananarivo Madagascar.

## Methods

A cross-sectional study was conducted based on a review of medical registries in the Department of Dermatology, University Hospital Joseph Raseta Befelatanana, Antananarivo Madagascar. Patients who required healthcare services from 2010 to 2016 were included. The inclusion criteria were age < 15 years, registered diagnosis of AD. AD was diagnosed by the reference dermatologist according to the criteria of United Kingdom Working Party modified. Age, sex, birth months, age of onset, associated comorbidities, breastfeeding duration, personal and familial past medical history (particularly personal and familial atopy) were obtained. Topographical distribution of lesions and body surface area were evaluated.

All study procedures were performed in accordance with the Ethics Committee of University Hospital Joseph Raseta Befelatanana Antananarivo, Madagascar. Study participants and their parents were informed about the study procedures and written informed consent was obtained.

Data collection was made by logiciel Microsoft Excel. Statistical analyses were processed by « logiciel R ». The X^2^ test was used to analyze the results, and p < 0.05 was considered statistically significant.

## Results

151 cases of atopic dermatitis were identified among 2665 children < 15 years seen in the Department of Dermatology, University Hospital Joseph Raseta Befelatanana Antananarivo Madagascar from 2010 to 2016, with the prevalence of 5.6%. 80 cases (52.9%) were < 2 years and 71 cases were > 2 years. The mean age of AD patients was 4 years. The mean age of onset was 3 years. The age of onset of AD in 104 children (69%) was before the age of 5 years 70.22%. There was a female preponderance (sex-ratio: 0.73). Distribution of participants via sociodemographic and clinical characteristics was shown in Table 1.

**Table 1:** Distribution of participants via sociodemographic and clinical characteristics

Characteristics	N	%
Sex		
Male	64	42.3
Female	87	57.6
Age group (years)		
0–2	66	43.7
2–5	33	21.8
5–10	36	23.8
10–15	16	10.6
Mean age: 4 Median: 2	Minimum: 6 months	Maximum: 14 years
Age of onset (years)		
0–5	105	69.5
5–10	33	21.8
10–15	13	8,6
Personal atopy		
Allergic rhinitis	32	21.1
Food allergy	25	16.5
Drug allergy	2	1.3
Atopic dermatitis	19	12.5
Asthma	9	5.9
Allergic conjonctivitis	5	3.3
Clinical presentation		
Acute lesions (papulovesicular lesions)	118	78.1
Impetiginized lesion	13	8.6
Lichenification	10	6.6
Body surface area (%)		
< 5	5	3.3
5–10	6	3.9
10–20	72	47.6
> 20	68	45

Children born in March (dry season) had the highest risk of AD (12.39% of cases). Concerning breast-feeding, 77% of children had exclusive or partial breast-feeding for at least 12 months. No association between breast-feeding and AD was found in our study. The correlation between the duration of breastfeeding and AD prevalence was shown in Table 2 (p = 0295). Consultations for AD increased during the winter (from July to October) (p = 0.005). Furthermore, the prevalence of AD was similar in urban (developed regions where pollution is more severe) and rural areas in our study. The frequency of AD consultation according to season and to geographical origin was shown also in Table 2.

**Table 2:** Correlation between the geographical origin, the duration of breastfeeding and the prevalence of AD

Characteristics	N	%	p value
Geographical origin			
Urban	75	49.67	0.89
Rural	76	50.33	
Breastfeeding duration (months)			
3–6	116	76.8	
6–12	31	20.5	0.295
> 12mois	4	2.6	
Season of consultation			
Winter (dry season: July to October)	78	51.6	0.005
Summer (rainy season: December to April)	33	21.8	

**Fig. 1:**
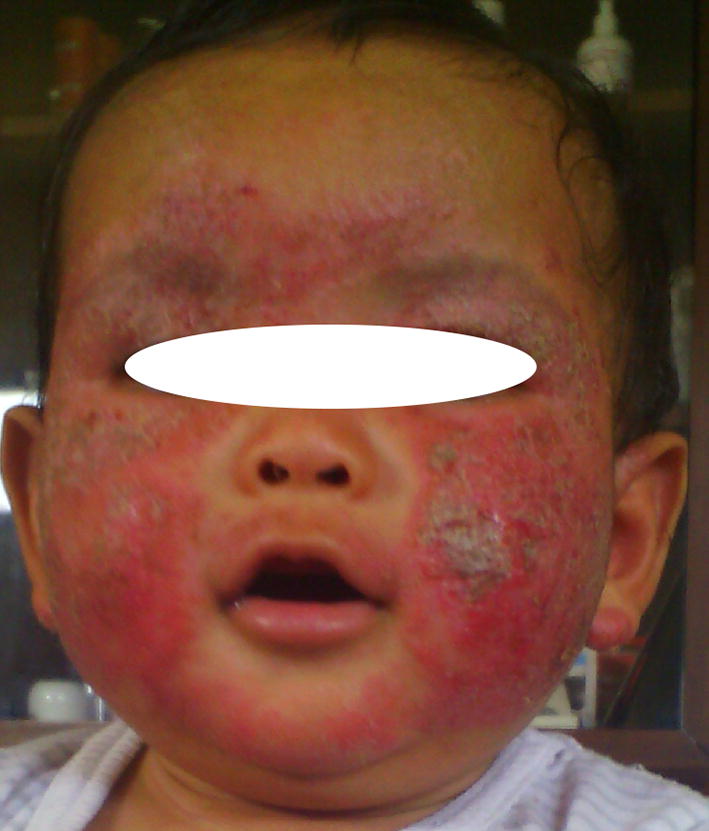
Facial involvement of atopic dermatitis

**Fig. 2:**
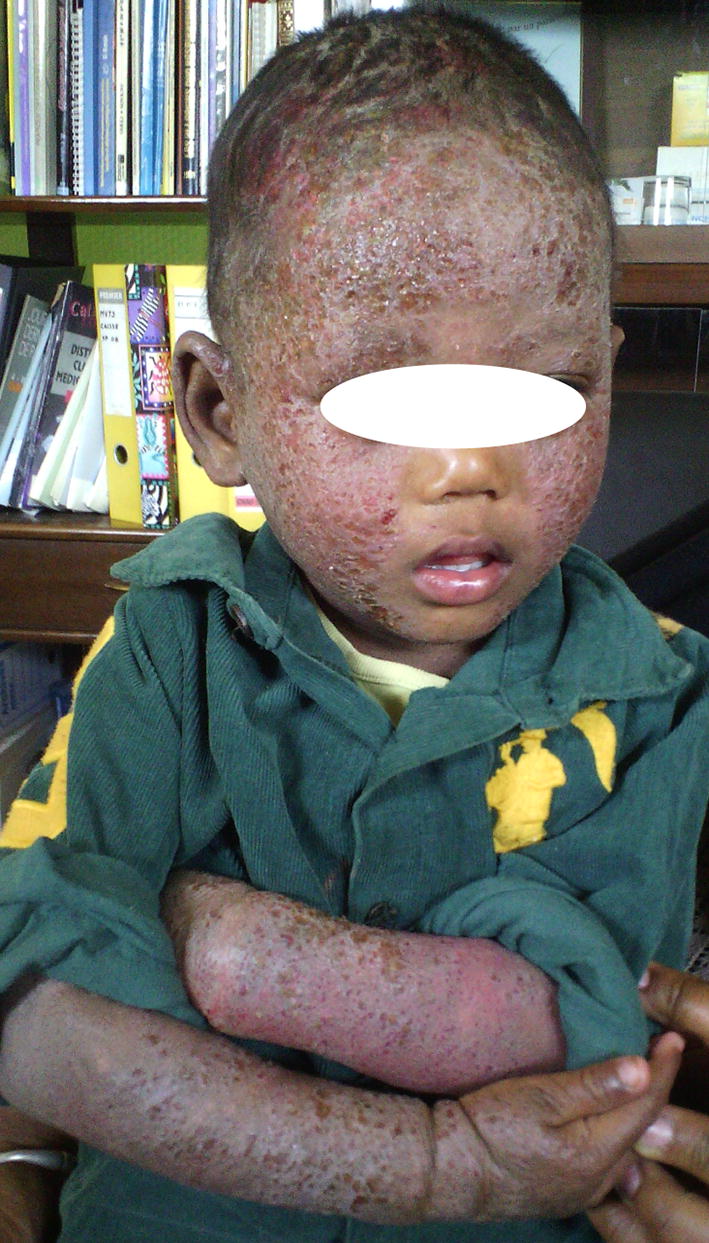
Extensive atopic dermatitis

**Fig. 3:**
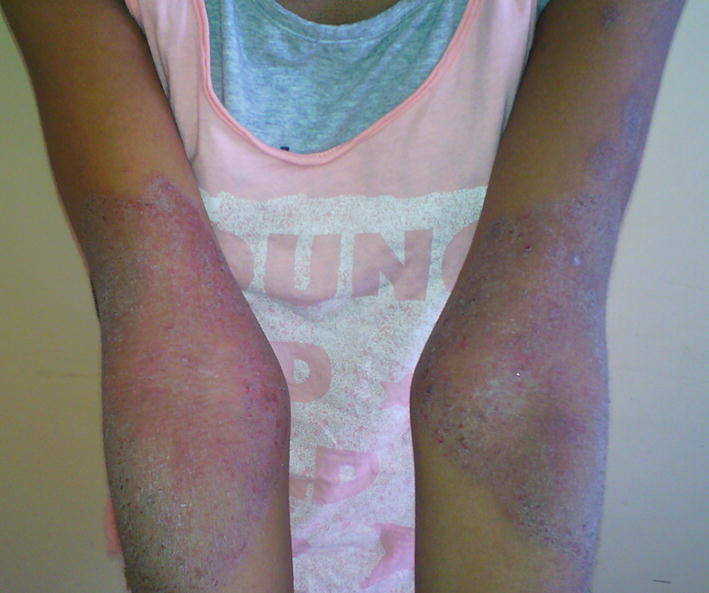
Atopic dermatitis on the flexures of the elbows

Pruritus and xerosis were present in 62.9 and 64.2% of cases, respectively. Topographical distribution by age was shown in Table 3. Photographs representing topographical distribution by age are shown in Figs. 1, 2 and 3. No association between body surface affected and pruritus was found (p value = 0.482).

**Table 3:** Topographical distribution by age

Topographical distribution of AD	Children < 2 years	Children > 2 years	p value
Convex areas of the face	51	32	0.014
Extensor surface of upper limb	30	25	0.45
Lower limb	21	22	0.9
Skin folds (neck, large flexures of the elbows and knees)	14	20	0.27
Scalp	9	2	0.03

Papulovesicular lesion was present in 101 cases (66.6%). Impetiginized and lichenified lesions were present in 13 (8.6%) and 4 cases (2.64%), respectively.

A family history of atopy was noted in 71 cases (47.01%), especially with family history AD in 56 cases (37%). Atopic dermatitis was associated with allergic rhinitis in 33 cases (21.8%), food allergy in 25 cases (16.5%) and asthma in 9 cases (5.9%).

## Discussion

This study reports the recent epidemiology of AD in children < 15 years in the Department of Dermatology, University Hospital Joseph Raseta Befelatanana Antananarivo Madagascar.

Our result shows that the prevalence of AD in children < 15 years (5.6%) has been raised in recent years, much higher than that from previous study (1.02% in a previous study in 3593 children) [5]. It is consistent with results from other studies. Atopic dermatitis in France has a prevalence of 6 to 9% before the age of 15, depending on the strictness of the criteria applied [2]. The « hygiene hypothesis » offers a potentially credible and parsimonious explanation for the increasing prevalence of atopic dermatitis noted in many westernized populations. The model of the hygiene theory has been clarified in recent years: environmental factors alter the diversity of skin and digestive microbiomes, and this diversity seems to play a major role in the development of atopy [6].

The results of some studies analyzing the relationship between urbanization and AD are different from ours. A Chinese study showed that the prevalence of AD in urban areas of Shanghai was gradiently and significantly higher than in rural areas (4.6% vs 10.2%) [7]. A marked urban–rural gradient was evident also in Ethiopian population, Yemaneberhan et al. reported that lifestyle factors linked to urbanization were associated with an increased risk of AD [8]. According to Kantor R, urban living may be associated with increased stress, greater proximity to automobile traffic and related pollutants [4].

The result of our study found an increased risk of AD in infants born during dry season (March to September). It is consistent with findings from some studies. Taiwanese case–control study reported that children born in October, November and December had higher risk of AD compared with children born in May. The relative humidity in October, November and December is relatively low in Taiwan [9]. A cross-sectional report in Japanese pediatric population showed that children born in the automn (October, November and December) were more likely to have AD [10]. One study who examined the association of season of birth with DNA Methylation and allergic disease found that autumn birth increased risk of eczema, relative to spring birth [11]. A meta-analysis including 9 studies in the northern hemisphere found also that AD was significantly associated with fall and winter compared with spring birth [12].

Some studies reported a different effect of breastfeeding on AD. A meta-analysis found that exclusive breastfeeding for at least 4 months can reduce the incidence of AD in infants [13]. However, new evidence from a 2016 cohort study now suggests that breastfeeding itself may be a risk factor. The study proposes that the breastfeeding timeline might be crucial in determining whether it prevents or incites the development of AD in all children regardless of risk [14]. A Japanese study reported also that the duration of exclusive breastfeeding for at least 6 months were associated with the prevalence of childhood eczema in a nationwide web survey [15]. Neither development of AD in children nor the age of onset of AD has correlation between the duration of breastfeeding in our study; this finding is consistent with other studies.

70 children (47%) had familial history of atopic diseases in our study. Our finding was lower than previous study reported by Wen HJ in which 70% of AD patients had a positive family history of atopic diseases [16]. Wadonda-Kabondo N et al. reported also that the odds of developing AD are 2 to threefold higher in children with one atopic parent, and this increases to three to fivefold if both parents are atopic [17]. Indeed, several genes were linked to the epidermal function and the immune system. The common loss-of-function variants within the fillagrin gene which encode the epidermal barrier protein fillagrin and their association with AD have important interest in the significance of skin barrier impairment in the development of AD [18]. Fillagrin determines the skin barrier integrity. Rehbinder EM et al. reported also that parental and pregnancy related factors (such as delivery > 38 gestational weeks, paternal age > 37 years, multiparity and maternal allergic diseases) were predictive for dry skin and AD [19].

## Conclusion

The present study shows that weather may have an impact on the prevalence of atopic dermatitis in Madagascar. No significant correlation is found between the duration of breastfeeding and AD, as well as urbanization.

## Data Availability

Not applicable.
